# Clinical reassessment of human embryo ploidy status between cleavage and blastocyst stage by Next Generation Sequencing

**DOI:** 10.1371/journal.pone.0201652

**Published:** 2018-08-22

**Authors:** Alberto Liñán, Barbara Lawrenz, Ibrahim El Khatib, Asina Bayram, Ana Arnanz, Carmen Rubio, Rupali Chopra, Human M. Fatemi

**Affiliations:** 1 IVF Laboratory, IVIRMA Middle-East Fertility Clinic, Abu Dhabi, United Arab Emirates; 2 IVF department, IVIRMA Middle East Fertility Clinic, Abu Dhabi, United Arab Emirates; 3 Obstetrical Department, Women´s University Hospital Tuebingen, Tuebingen, Germany; 4 Igenomix, Valencia, Spain; 5 Igenomix, Dubai, UAE; Peking University Third Hospital, CHINA

## Abstract

One of the most important limitations of genetic testing in preimplantation embryos is embryonic mosaicism, especially when performed on D3 with only a single blastomere evaluated. Previous publications, using Array-Comparative Genomic Hybridization (a-CGH) to compare day 3 (D3) biopsies versus trophectoderm biopsies for the analysis of aneuploid embryos, showed similar high concordance rates per embryo diagnosis for D3 biopsies and trophectoderm biopsies. Next generation sequencing (NGS) was introduced lately as a new technique for preimplantation genetic testing for aneuploidies (PGT-A). Using this technique, this retrospective descriptive study evaluated the degree of the concordance of the diagnosis between preimplantation human cleavage stage (D3) and blastocyst stage (D5) embryos. Double biopsies on D3 and D5 were performed on 118 embryos, reaching blastocyst stage on D5 and had not been selected for transfer. As the fertilization law of the United Arab Emirates does not allow embryo freezing, also surplus euploid embryos after D 3 biopsy were included.

Analysis of the NGS results from D3 and D5 embryo biopsies showed a total concordance rate per embryo diagnosis of 85.6% for euploid and aneuploid embryos. The concordance rates per embryo chromosomal pattern for embryo diagnosed as aneuploid at both biopsy stages was 82.2%. However, the status regarding the affected chromosomes was not identical on D3 and D5. Hence, the total concordance rate between D3 biopsy and D5 biopsy was limited to 67.8%.

This current study clearly demonstrated that the concordance rates between D3 and D5 biopsies in aneuploid and euploid embryos are lower than previously reported.

## Introduction

Infertility is a common condition today [1] and exacerbated by the fact that couples tend to postpone parenthood, leading to an increasing age in couples attempting to conceive [2]. Since the first successful IVF treatment with the birth of Louise Joy Brown in 1978, assisted reproductive technologies (ART) have improved the chances of infertile couples to achieve a pregnancy [3].

Despite the improvements in ART, success rates are still limited [4,5]. One of the most crucial factors determining success of ART-treatment is the female age, due to increasing aneuploidy rates in oocytes in women of advanced age [6]. In addition to female age, also male age has recently been identified as a determinant of delivery rates after ART [7], probably due to factors such as age-dependent increase of sperm DNA-damage [8]. Other factors, being relevant for aneuploidy in human spermatozoa are the severity of male factor infertility, abnormal karyotype of the male partner and it seems that also lifestyle factors like smoking, alcohol consum and exposure to pesticides might contribute to aneuploidy in human spermatozoa [9]. Nevertheless, the detection of chromosomal abnormality rates up to 62.9% in couples with a mean female age of 32.33 years suggest that these aneuploidies are not exclusively attributed to advanced maternal age [10,11].

The most important cause for implantation failure is chromosomal abnormality in the embryo [12,13]. Standard morphological criteria and development pattern cannot predict the ploidy status of embryos [14] and the only reliable method for aneuploidy detection requires biopsy of the embryo and subsequent analysis of the chromosomal status.

Genetic testing of the embryo has led to the first reported pregnancy in 1995 after the use of fluorescence in situ hybridization technique (FISH) [15]. Newer technologies have displaced the FISH-technique, considering that only a limited number of chromosomes could be evaluated, missing approximately up to 1/3 of aneuploidies [16]. Nowadays analysis of the whole chromosome complement is performed with different genetic platforms like metaphase comparative genomic hybridization (mCGH), array comparative genomic hybridization (aCGH), single nucleotide polymorphism (SNP) microarray, quantitative polymerase chain reaction (qPCR), and most recently, Next Generation Sequencing (NGS) [17–21].

In general, embryos can be assessed by testing single blastomeres from cleavage stage embryos or by analysis of several cells from the trophectoderm at blastocyst stage. Despite the different approaches, embryonic mosaicism is an important limitation of embryo biopsy [22,23] and it has been described extensively, that mosaicism is present and common during preimplantation development [24]. If aneuploidy is detected with PGT-A, the embryo is deemed abnormal. Hence, due to mosaicism, the discarding of potentially viable, euploid embryos might occur.

The aim of this descriptive study was to evaluate the extend of the concordance / discordance between the diagnosis in cleavage stage and blastocyst stage embryos, which may occur as a result of mosaicism or technical error. For that purpose, we re-evaluated the NGS findings after cleavage stage embryo biopsies by re-analysing the same embryos with trophectoderm biopsy. To the best of our knowledge this is the only study which could not only re-evaluate the results from aneuploid embryos, hence also from euploid embryos. This was possible due to the law of the United Arab Emirates (UAE) governing ART which does not permit embryo freezing [25] as a routine procedure. As a result of this law, surplus euploid embryos cannot be cryopreserved for future transfer and as a result are discarded.

## Material and methods

### Patients

Embryos of couples with primary or secondary infertility requiring IVF/ICSI and additional Preimplantation Genetic Testing (PGT) were included in this descriptive study. The age of the female partner ranged between 20 to 46 years and the Body Mass Index of the female partner between 19 and 30. Embryos of patients, in whom more than 5 fresh, mature oocytes were retrieved at oocyte collection or more than 4 embryos were available for biopsy on D3, were included.

### Study design

This retrospective descriptive study was performed in IVIRMA Middle East Fertility Clinic, Abu Dhabi, from August 2016 to January 2017. Biopsies of the embryos had been performed on day 3 (D3) and day 5 (D5) for confirmation of the genetic results. The best euploid embryo(s) after biopsy on D3 and assessment of embryo development and blastocyst score had been chosen for transfer. Surplus euploid blastocysts, which had not been selected for transfer and were not cryopreserved in accordance with UAE law had been included. The genetic laboratory was blinded regarding the embryo identity.

#### Ovarian stimulation protocols

Ovarian stimulation was performed by standard protocols, either Gonadotropin-Releasing-Hormone (GnRH)-agonist- or GnRH-antagonist-protocols, using recFSH (recombinant Follicle-stimulating-hormone) or human-Menopausal-Gonadotropin (HMG) as stimulation medication. The dosage of the stimulation medication was chosen according to the ovarian reserve parameters [26]. Final oocyte maturation was achieved by administration of either 10.000 IU of hCG, 0.3 mg of GnRH agonist (Triptorelin) or dual trigger (hCG and GnRH-analogue), as soon as ≥ 3 follicles ≥ 17 mm were present. Oocyte retrieval was carried out 36 hours after administration of final oocyte maturation.

#### Embryo processing and embryo biopsy

Fertilization was assessed about 17–20 h post ICSI, and embryo development was recorded every 24 h until the day of embryo transfer. Embryos were cultured in Quinn’s Advantage Sequential medium, (SAGE, Målov, Denmark), using Trigas incubators (6%CO2, 5%O2).

D3 embryo biopsy was only performed in embryos with five or more nucleated blastomeres and less than 25% fragmentation degree. Embryos were placed on a droplet containing Ca2+/Mg2+-free medium (G-PGD, Vitrolife, Göteborg, Sweden/LifeGlobal, Guilford, CT), the zona pellucida was perforated by pulses of laser (OCTAX, Herborn, Germany) and one blastomere was withdrawn from each embryo and placed in 0.2-mL PCR tubes containing 2 μL PBS. For blastomere washing and handling, 1% polyvinylpyrrolidone (PVP) was used.

For trophectoderm biopsy, embryos were biopsied using Quinn’s Advantage Medium with HEPES, (SAGE, Målov, Denmark) supplemented with HSA, (Vitrolife, Göteborg, Sweden). Three to five laser pulses were used to cut the trophectoderm cells inside the aspiration pipette and then trophectoderm biopsies were placed in 0.2-ml PCR tubes containing 2 μL PBS.

#### Determination of aneuploidy by NGS

The herein used NGS platform has been validated in previous studies [27,28] and is commercially available in the market. This platform has been used to analyse blastomere biopsies and trophectoderm biopsies, (Resproseq, Life-Thermofisher, USA).

A whole genome amplification (WGA) protocol was performed in all individual samples. (PicoPlex technology by Rubicon Genomics,Inc; Ann Arbor,Michigan, USA.). After WGA, library preparation consisted on the incorporation of individual barcodes for the amplified DNA of each embryo. After Isothermal amplification and enrichment, sequencing was performed in a 316 or 318 chip using the PGM sequencing machine (Life-Thermofisher, USA). For sequencing analysis and data interpretation Ion Reporter software was employed. Embryos were diagnosed as euploid, aneuploid or chaotic abnormal. In case of a result indicating mosaicism, the embryo was classified as “euploid” when the extend of mosaicism was below 30% and as “aneuploid” when the extend of mosaicism was above 30%. Chaotic embryos were defined as those showing a complex pattern of aneuploidies, involving more than six chromosomes.

#### Concordance/Discordance rates

Discrepancy is described as a lack of agreement between two or more facts, i.e. aneuploid on D3 and euploid on D5 or vice versa. The genetic definition of discordance determines the degree of dissimilarity between the chromosomes evaluated.

Herein we have included as discrepant results not only the euploid embryos on D3 that were aneuploid on D5 and vice versa, hence also the aneuploid embryos on D3, that were still aneuploid on D5, but with different chromosomes involved in the aneuploidy. For the calculations of the “false positive” and “false negative” rates, we have chosen the blastocyst stage biopsy as the reference, considering the higher technical and biological robustness of trophectoderm biopy, the decreased incidence of procedural errors and lower impact of mosaicism on the molecular analysis with day 5 trophectoderm biopsy [29].

#### Calculation of the concordance / discordance rates

Two types of concordance rates had been calculated: the concordance rate per analysed chromosome, where the total number of chromosomes was considered, independently whether the embryos were euploid or aneuploid (24 chromosomes per embryo) and the concordance rate per embryo diagnosis, where discrepancies in the embryo diagnosis (euploid or aneuploid) were considered. Concordance / discordance rates have been calculated as previously described by Vera-Rodriguez et al. [30].

### Statistical analysis

For the statistical analysis of the Confidence Intervall (CI), the 95% Confidence Interval calculator for proportions has been applied.

#### Ethical approval

Written informed consent was obtained from the couples, whose embryos were undergoing biopsies on day 3 and day 5. The study was approved by the Ethic Committee of IVIRMA Middle East Fertility Clinic, Abu Dhabi, UAE (Research Ethics Committee IVI-MEREF010/2017).

## Results

In this study, initial data of 134 embryos from 45 couples were analysed. The mean age of the female partners was 33.4 years, ranging from 20 to 46 years, and the mean number of biopsied embryos per couple was 4.4, with a range from 1 to 9. The indications to perform genetic testing on the embryos are summarized in Table 1.

**Table 1:** Indications for Preimplantation Genetic Testing for Aneuploidy (PGT-A).

Indications for PGT-A	Number of couples	Number of embryos
**Advanced maternal age (AMA)**	13 (31%)	43 (36.4%)
**Severe male factor**	9 (21.4%)	23 (19.5%)
**Recurrent implantation failure**	5 (11.9%)	18 (15.3%)
**Recurrent miscarriage**	6 (14.3%)	14 (11.9)
**Previous aneuploidy conception**	2 (4.8%)	5 (4.2%)
**Elective aneuploidy screening**	7 (16.6%)	15 (12.7%)
**TOTAL NUMBER**	42	118

Out of the 134 embryos analysed on D3, 6 embryos with “complex abnormal” result and 5 embryos that failed to produce a result due to amplification failure (no DNA was detected after cleavage stage biopsy) were excluded from the study. Out of these 6 embryos with complex abnormal findings on cleavage stage, 3 were euploid after blastocyst biopsy. After blastocyst stage biopsy, five embryos were excluded due to amplification failure. Finally a total of 118 embryos from 42 couples were included and biopsies on D3 and D5 were performed. Table 2 summarizes the data of the analysed embryos.

**Table 2:** Summary of the analysed embryos.

	BIOPSY D3	BIOPSY D5
No. of embryos analysed	134	134
No. of informative embryos	129 (96.3%)	129 (96.3%)
No. of “No DNA detected” embryos	5 (3.7%)	5 (3.7%)
No. of complex abnormal embryos	6 (4.5%)	0
No. of embryos included in the study	118 (100%)	
No. of euploid embryos (%)	48 (40.7%)	63 (53.4%)
No. of aneuploid embryos (%)	70 (59.3%)	55 (46.6%)
No. of euploid D3—aneuploid D5 **(false negative)** Confidence Intervall (CI)	1/118 (0.85%) 95% CI: 0–2.50%	
No. of aneuploid D3-euploid D5 **(false positive)** Confidence Intervall (CI)	16/118 (13.6%) 95% CI: 7.38–19.74%	
No. of whole chromosome aneuploid D3—euploid D5 Confidence Intervall (CI)	9/118 (7.6%) 95% CI: 2.84–12.42%	
No.of segmental chromosome aneuploid D3-euploid D5 Confidence Intervall (CI)	7/118 (5.9%) 95% CI:1.67–10.19%	

### Concordance / Discordance rates

The overall concordance rate per analysed chromosome (24 chromosomes x 118 embryos) was 98.1% (2779/2832 analysed chromosomes). For whole-chromosome aneuploidy, the concordance rate was 98.7% (2795/2832) and for segmental aneuploidies was 99.4% (2816/2832). The concordance rate for whole-chromosome aneuploidies per embryo diagnosis (euploid/aneuploid) was 91.5% (108/118) and 94.1% (111/118) for segmental aneuploidies, leading to a total concordance rate per embryo diagnosis of 85.6% (101/118). The data are summarized in Table 3.

**Table 3:** Concordance rates per embryo diagnosis (euploid / aneuploid) and for segmental and whole chromosome aneuploidies per chromosome.

	Concordance rate per embyo diagnosis (euploid / aneuploid) (Percentage and number of embryos)	Concordance rates for segmental and whole chromosome aneuploidies per chromosome. (Percentage and number of chromosomes)
**Overall concordance rate** **Confidence interval**	**85.6%** (101/118) 95%CI: 79.26–91.93%	**98.1%** (2779/2832) 95%CI: 97.63–98.63%
**Whole chromosome aneuploidy** **Confidence Interval**	**91.5%** (108/118) 95%CI: 86.50–96.55%	**98.7%** (2795/2832) 95%CI: 98.28–99.11%
**Segmental aneuploidy** **Confidence Interval**	**94.1%** (111/118) 95%CI: 89.81–98.33%	**99.4%** (2816/2832) 95% CI: 99.16–99.71%

CI = Confidence interval.

Per embryo diagnosis, 85.6% (95%CI: 79.26–91.93%) of the embryos were concordant, i.e. they had the same diagnosis (euploid or aneuploid) at cleavage and trophectoderm biopsy. However, 17 (14.4%; 95%CI: 8.07–20.74%) discrepancies per embryo diagnosis were found. Table 4 summarizes the data.

**Table 4:** Summary of the discrepancies per embryo diagnosis.

BIOPSY D3	BIOPSY D5
Abnormal: -1, -8, ♀	Normal: ♀
Abnormal: +12p, ♂	Normal: ♂
Abnormal: -2p ♂	Normal: ♂
Abnormal: +19 ♀	Normal: ♀
Abnormal: -12 ♀	Normal: ♀
Abnormal: +9q ♀	Normal: ♀
Abnormal: -20 XXY	Normal: ♂
Abnormal: +10 ♂	Normal: ♂
Abnormal: -2q ♂	Normal: ♂
Abnormal: -4 ♀	Normal: ♀
Abnormal: +11p ♂	Normal: ♂
Abnormal: +17 ♂	Normal: ♂
Abnormal: +14 ♂	Normal: ♂
Abnormal: +10p ♂	Normal: ♂
Normal: ♀	Abnormal: -12, -13, +21, ♀
Abnormal: -6q ♀	Normal: ♀
Abnormal: +19 ♀	Normal: ♀

The false positive rate per embryo diagnosis, i.e. embryos, which were initially diagnosed as aneuploid on D3 were diagnosed as euploid embryos on D5 and hence transferable, was 13.6% (16 out of 118 embryos). Out of these 16 embryos, 7.6% (9/118) were whole-chromosome discrepancies and 5.9% (7/118) were segmental aneuploidies. False negative rate per embryo diagnosis, i.e. embryos being diagnosed as euploid on D3 and being diagnosed as aneuploid on D5, was 0.85% (1 out of 118 embryos, see Table 2).

### Concordance rate for aneuploid embryos

The concordance rate for the chromosomal pattern in aneuploid D3 / aneuploid D5 embryos was 82.2% (97/118) (95%CI: 75.30–89.10%), representing embryos aneuploid at both biopsy stages. However, the chromosomal status regarding the affected chromosomes was not identical on D3 and D5. 14 embryos had whole chromosomes discrepancies and 7 embryos had segmental discrepancies. The discrepant chromosomes in both embryo stages are represented in Table 5.

**Table 5:** Discordance-rate per chromosome (Aneuploid D3-Aneuploid D5).

D3 PGS diagnosis	D5 PGS diagnosis	No of discrepant chromosomes	Segmental discrepancy Yes / No
Abnormal: -5, -11, -13, ♀	Abnormal: -5p, ♀	2	N
Abnormal: +6, -19, ♀	ABnormal: -19, ♀	1	N
Abnormal: X0	Abnormal: +Xp, (XXX)	1	N
Abnormal: +6, +16, +22, ♂	Abnormal: +6, +16, -18, +22, ♂	1	N
Abnormal: -19, +21, +22, ♂	Abnormal: +21, +22, ♂	1	N
Abnormal: -3, +6, -22, ♀	Abnormal: +3, +4, +6, -22, ♀	1	N
Abnormal: -13, +16, ♀	Abnormal: +16, ♀	1	N
Abnormal: -4q, +21, ♀	Abnormal: +7, +10, +16, +18, +20, ♀	7	N
Abnormal: -3q, ♂	Abnormal: -2q, ♂	1	Y
Abnormal: +12p, +21, ♀	Abnormal: +21, ♀	1	Y
Abnormal: -8, -11q, ♀	Abnormal: -8, ♀	1	Y
Abnormal: +16, ♂	Abnormal: -1q, +8, +16, ♂	2	N
Abnormal: -13p, ♂	Abnormal: +1q, +7p, ♂	3	Y
Abnormal: +8, ♀	Abnormal: +8, -19, ♀	1	N
Abnormal: -22, ♂	Abnormal: -7q, -22, ♂	1	Y
Abnormal: +15, +16, -20, X0	Abnormal: +15, +16, +19, -20, X0	1	N
Abnormal: +2q, -10, ♀	Abnormal: -2q, -13, ♀	2	N
Abnormal: +2, -22, ♀	Abnormal: -22, ♀	1	N
Abnormal: +1, +4p, ♀	Abnormal: +1, ♀	1	Y
Abnormal: -9, +14, -15, -21, ♀	Abnormal: -9, +14, -15, ♀	1	N
Abnormal: +5q, -16, ♂	Abnormal: -16, ♂	1	Y

Out of 70 aneuploid embryos on D3, 16 (22.9%; 95% CI: 13.02–32.69%) showed a euploid status after blastocyst biopsy. Fifty-four embryos were aneuploid after biopsy on D3 and D5. In depth analysis of these 54 embryos 33 (61.1%; 95% CI: 48.11–74.11%) were found to be concordant for all the aneuploid chromosomes. Twenty-one embryos (38.9%; 95% CI: 25.89–51.89%) had discrepant chromosomes with different kinds of aneuploidy on D3 and/or D5.

### Concordance rate for euploid embryos

The analysis of D3 euploid embryos showed that 47 out of 48 (97.9%; 95%CI: 93.88–101.96%) were diagnosed as euploid on D5 as well. The embryo, which was not concordant, showed an aneuploid chromosomal constitution (-12–13 +21 ♀) on D 5.

### Overall concordance rate

The total rate of discrepancies was 32.2% (95% CI: 23.77–40.63%), composed of 13.6% (False positive) + 0.85% (False negative) +17.8% (aneuploid D3-aneuploid D5, with different aneuploidies either on D3 or D5). Hence the total concordance rate between D3 biopsy and D5 biopsy was limited to only 67.8%.

## Discussion

To the best of our knowledge, this is the first study re-evaluating not only aneuploid, but also euploid embryos with double biopsy on D3 and D5 using NGS technology. This is unique due to the law governing ART in the United Arab Emirates, which does not permit the cryopreservation of surplus embryos routinely.

Contrary to previous publications, which reported higher concordance rates between blastomere and trophectoderm biopsies with aCGH technology [31], the current descriptive study, which includes 118 embryos from 42 couples revealed a total concordance rate per embryo diagnosis (euploid/aneuploid) of 85.6%. This concordance rate of 85.6% represents embryos with the same diagnosis following both cleavage stage and trophectoderm biopsy. In 14.4% (17 embryos) discrepancies per embryo diagnosis (euploid/aneuploid) were detected. However, when the discrepancies between the diagnosis of the aneuploid embryos were considered (different chromosomes involved in the aneuploidy between D 3 and D 5 biopsy), the total discrepancy rate was as high as 32.2%.

The false positive rate per embryo diagnosis, meaning that embryos, which were initially diagnosed as aneuploid on D3 were finally diagnosed as euploid embryos on D5, was 13.6%. This finding underlines the decrease of aneuploidy from cleavage to blastocyst stage embryo, which is in agreement with the data from Fragouli et al. [32], who reported an aneuploidy rate of 83% in cleavage stage embryos and 58% in blastocyst stage embryos. However, Fragouli et al. [32] compared the aneuploidy rate, detected with microarray comparative genomic hybridisation, in embryos biopsied at cleavage stage and embryos at blastocyst stage, which were derived from different couples and did not re-biopsy the same embryo, as performed in the herein presented data.

The biological explanation for the discrepancies in this study might be potential mitotic errors in the division of the blastomeres during the embryo development, giving rise to mosaic embryos with euploid and aneuploid blastomeres [33]. Contrary to meiotic errors, mitotic errors are not consistent with maternal age, showing only weak propensities for specific chromosomes, and often affect many chromosomes simultaneously [34]. However, also amplification artefacts from single cell genetic technologies on D3 biopsies could be the underlying cause for discrepancies, leading to a high percentage of false positive rate per embryo diagnosis [35]. A previous study by Capalbo et al [36] identified a sensitivity of 86.4% and a specificity of 95% with D3 biopsy with the use of aCGH technique. Compared to aCGH-technigue, NGS technique allows identification of embryos with chromosomal mosaicism and segmental aneuploidy more precisely than the PGS/aCGH platform [37] and this fact may account partly for the lower concordance rates observed in our study, compared to the previous aCGH studies above mentioned.

The topic of discrepant results of genetic testing between cleavage and blastocyst stage embryos with a decline in the aneuploidy rate towards blastocyst stage has been addressed by several studies. The main limitation of the first publications, which described inconsistent results between cleavage and blastocyst stages, is the use of FISH (Fluorescent in situ hybridization)–technique [38–40]. Later on, other studies confirmed the discrepant results by the use of different techniques [32,41–44] and those findings are in agreement with our results.

The following mechanisms have been suggested for the reduced rate of aneuploidy in blastocyst stage embryos: the occurrence of cell arrest or apoptosis of the aneuploid blastomeres, the active correction or self-correction of the aneuploidy and the allocation of diploid/aneuploid blastomeres to embryonic or extra-embryonic tissues. Moreover, the possibility of primary misdiagnosis has to be considered [45,46]. Cell arrest is initiated after the time of embryonic genome activation and seems to prevent chromosomal abnormal blastomeres from further development [46]. Additionally, apoptosis of aneuploidy blastomeres could also be initiated by weaker mitotic checkpoints [47]. Finally the activation of apoptosis of aneuploid blastomeres in the blastocyst would result in embryos with a higher proportion of euploid cells [48]. Self-correction has been observed in uniparental disomy, however the exact mechanism is not known. Theoretically, mechanism like anaphase lagging or non-disjunction, which might cause mitotic errors, might be also able to correct them [40]. The finding of aneuploid cells only in the placenta and not in the embryo is the basis of the theory of “preferential allocation”, assuming that aneuploid blastomeres are allocated to the trophectoderm where the detrimental impact of the aneuploidy is lower [49]. However, presence of a similar degree of mosaicism in the inner cell mass when compared to trophectoderm, does not support the idea of preferential allocation of aneuploid blastomeres.

For the correct interpretation of the results from genetic testing on D3 and D5, the biological and technical limitations have to be considered [50]. Due to the highly variable rate of mosaicism in cleavage-stage embryos [15,51–53]. D3 biopsies for PGT-A have been criticized for not being the optimal and accurate technique for ploidy screening and trophectoderm biopsy is considered to be the more reliable technique [50]. However, a recently published study showed similar concordance rates with whole blastocyst results on D5, performed as re-analysis on embryos with previous D3 biopsy [31].

Presence of mosaicism in the embryo might pose an important factor for the observed discordance rate found in this study. Four different types of mosaic embryos have been described at blastocyst stage, according to the cell lineage affected: Total mosaic, inner cell mass (ICM) mosaic, trophectoderm (TE) mosaic and ICM/TE mosaic [23]. The different types of mosaicism might make it impossible to detect some of the mosaic embryos with a trophectoderm biopsy, especially as ICM/TE and ICM mosaicism will never be detectable with the use of a trophectoderm biopsy. Furthermore, the diagnosis of TE mosaic embryos will differ, depending on the biopsy location of the TE cells [23]. Therefore the factors mosaicism type and degree, biopsy location, number of cells biopsied and quality of the biopsy sample are critical regarding the discrepancies between blastocyst and cleavage stage embryos.

Contrary to cleavage stage biopsy, trophectoderm biopsies contain multiple cells and therefore an increased number of copies of DNA material in the biopsy specimen. This provides greater fidelity and may reduce the non-result rate [54–56]. Consequently, a lower rate for mosaicism is expected with the approach of trophectoderm biopsy [50].

The predictive value of a TE biopsy to identify a mosaic embryo was evaluated by two studies. Two to three biopsies in the same embryo were performed and high concordance (95%-100%) were found [56–57]. In those studies also ICM from the same embryos were analysed to re-evaluate the concordance rate between ICM and TE. A discordant mosaicism rate of approximately 3%-4% was shown and based on this findings, blastocyst biopsy was confirmed as a valid method to diagnose blastocyst mosaicism accurately.

The limitations of this study are the retrospective and descriptive character and a possible bias regarding the concordance/discordance rate due to the fact, that embryos, which had been selected for transfer, did not undergo biopsy on day 5. The embryos had been selected according to the results of the genetic testing, done on day 3 and in case of several euploid embryos, according to morphology scoring. The importance of our findings might be scaled down by the increasing use of blastocyst biopsies instead of cleavage stage embryo biopsies, especially as cleavage stage biopsy can have an impact on future developmental and implantation potential. Despite this circumstances the current study clearly demonstrated that the concordance rates between D3 and D5 biopsies in aneuploid and euploid embryos are lower than previously reported. Future studies, including inner cell mass biopsies are required to evaluate the aetiology of the observed discrepancies between trophectoderm and blastomeres.
